# TMS-induced phase resets depend on TMS intensity and EEG phase

**DOI:** 10.1088/1741-2552/ad7f87

**Published:** 2024-10-24

**Authors:** Brian Erickson, Brian Kim, Philip Sabes, Ryan Rich, Abigail Hatcher, Guadalupe Fernandez-Nuñez, Georgios Mentzelopoulos, Flavia Vitale, John Medaglia

**Affiliations:** 1 Applied Cognitive and Brain Sciences, Department of Psychology, Drexel University, Philadelphia, PA 19104, United States of America; 2 Starfish Neuroscience, Bellevue, WA 98004, United States of America; 3 Department of Physiology, University of California, San Francisco, CA 94143, United States of America; 4 Department of Bioengineering, University of Pennsylvania, Philadelphia, PA 19104, United States of America; 5 Center for Neuroengineering and Therapeutics, University of Pennsylvania, Philadelphia, PA 19104, United States of America; 6 Center for Neurotrauma, Neurodegeneration, and Restoration, Corporal Michael J. Crescenz Veterans Affairs Medical Center, Philadelphia, PA 19104, United States of America; 7 Department of Neurology, University of Pennsylvania, Philadelphia, PA 19104, United States of America; 8 Department of Physical Medicine and Rehabilitation, University of Pennsylvania, Philadelphia, PA 19104, United States of America; 9 Department of Neurology, Drexel University, Philadelphia, PA 19104, United States of America

**Keywords:** electroencephalography, transcranial magnetic stimulation, phase resetting, motor cortex, transcranial evoked potential, phase dependence

## Abstract

*Objective*. The phase of the electroencephalographic (EEG) signal predicts performance in motor, somatosensory, and cognitive functions. Studies suggest that brain phase resets align neural oscillations with external stimuli, or couple oscillations across frequency bands and brain regions. Transcranial Magnetic Stimulation (TMS) can cause phase resets noninvasively in the cortex, thus providing the potential to control phase-sensitive cognitive functions. However, the relationship between TMS parameters and phase resetting is not fully understood. This is especially true of TMS intensity, which may be crucial to enabling precise control over the amount of phase resetting that is induced. Additionally, TMS phase resetting may interact with the instantaneous phase of the brain. Understanding these relationships is crucial to the development of more powerful and controllable stimulation protocols. *Approach.* To test these relationships, we conducted a TMS-EEG study. We applied single-pulse TMS at varying degrees of stimulation intensity to the motor area in an open loop. Offline, we used an autoregressive algorithm to estimate the phase of the intrinsic *µ*-Alpha rhythm of the motor cortex at the moment each TMS pulse was delivered. *Main results*. We identified post-stimulation epochs where *µ*-Alpha phase resetting and N100 amplitude depend parametrically on TMS intensity and are significant *versus* peripheral auditory sham stimulation. We observed *µ*-Alpha phase inversion after stimulations near peaks but not troughs in the endogenous *µ*-Alpha rhythm. *Significance*. These data suggest that low-intensity TMS primarily resets existing oscillations, while at higher intensities TMS may activate previously silent neurons, but only when endogenous oscillations are near the peak phase. These data can guide future studies that seek to induce phase resetting, and point to a way to manipulate the phase resetting effect of TMS by varying only the timing of the pulse with respect to ongoing brain activity.

## 1. Introduction

Brain oscillations are important in many cognitive functions (Lakatos *et al* 2019). In part, this is because the phase of the oscillatory cycle influences cortical excitability, which affects how likely regions are to process incoming stimuli (Schalk 2015). For instance, perception of a weak visual stimulus is enhanced when it aligns with the high cortical excitability period of the electroencephalogram (EEG; Busch *et al* 2009, Reinhart and Nguyen 2019). The brain uses the same mechanism to synchronize the receptive periods of different regions in order to facilitate long-distance communication (Fries 2005, Canavier 2015). The causal role of EEG is still a topic of debate (Engel and Gerloff 2022), but EEG phase at least tracks cortical excitability fluctuations that are broadly relevant to brain function.

To align the excitability-shaping properties of oscillations, the brain uses phase resets (Canavier 2015). Resetting has been studied invasively (Galán *et al* 2005, Tateno and Robinson 2007, Bauer *et al* 2020) and utilized in deep brain stimulation therapy for Parkinson’s and other diseases (Manos *et al* 2018). EEG phase resets are predictive of neural phase resets (Nuñez and Buño 2021, Kienitz *et al* 2022) and have been linked to behavior in tasks including motor, perception, attention, decision making and working memory (VanRullen 2016, 2018, Rawls *et al* 2020, Hussain *et al* 2021, Nakatani *et al* 2021, Wischnewski *et al* 2022, Mentzelopoulos *et al* 2023).

Transcranial Magnetic Stimulation (TMS) is a promising avenue for controlling phase resets. TMS has been used to modulate phase-sensitive functions (Paus *et al* 2001, Kienitz *et al* 2022) and TMS induced phase resets may be a factor in driving Transcranial-Evoked Potential components (TEPs; Makeig *et al* 2004, Kawasaki *et al* 2014). Between low and near-threshold TMS intensities must lie a transition zone where pyramidal neurons begin to activate. It seems likely that rTMS may have qualitatively different effects depending on how and which populations it activates. Many repetitive TMS (rTMS) protocols are delivered at sub-threshold intensities that may fall within this transition zone, but do not have strong theoretical justification for their choice of stimulation intensity. This is likely in part because the full parametric relationship between intensity and resetting has not been explored. Furthermore, the instantaneous phase of EEG influences how TMS affects the brain. At least one report found this to be true for late-epoch phase resets (Desideri *et al* 2019), but to our knowledge, these effects have only been studied at a few near-threshold intensities. Controlling phase resets will require a detailed understanding of how TMS parameters interact with endogenous state.

To address these fundamental issues, we measured TMS-induced phase resetting at intensities from 10% to 100% Resting Motor Threshold (RMT). After the session, we used an off-line predictive algorithm to estimate EEG phase at the time of stimulation (Zrenner *et al* 2018). We used these estimates to sort trials according to whether they occurred closer to a peak or trough to observe how phase resetting depends on brain state. We employed an auditory sham and active control condition to estimate peripheral effects. We expected that increasing TMS stimulation intensity would parametrically induce stronger phase resetting in both broadband and *µ*-Alpha filtered signals (the intrinsic rhythm of the motor cortex). We also expected an increase in N100 component amplitude (a proposed TEP marker of cortical excitability) and associated phase resetting above auditory sham and parietal control stimulation in both broadband and Alpha-band.

## 2. Methods

### 2.1. Participants

19 participants (6 male, 13 female; mean age = 23.9, SD = 6.1 years) were recruited through flyers and re-contacting. Written informed consent was obtained before participation and the study was conducted in accordance with the Declaration of Helsinki. Participants were screened for criteria including common TMS risk factors. All participants passed screening and were right-handed based on the Edinburgh Handedness Inventory (Oldfield, 1971; mean = 4.4, SD = 0.31; scores near 5 indicate right-handedness). Sessions lasted approximately two and a half hours. Participants were compensated $25/h for their time.

The presence of a *µ*-Alpha signal is critical for accurate phase estimation. As a result, we computed spectrograms for each participant across the whole recording and looked for a positive signal-to-noise ratio in the Alpha range, defined as significant alpha power above a regression fitted to adjacent frequencies in the power spectrum (Zrenner *et al* 2020). Seven participants were excluded due to a lack of a strong *µ*-Alpha signal, leaving 13 participants for analysis.

### 2.2. Experimental protocol

Participants were semi-reclined with a headrest and fitted with earplugs. A Brainsight neuronavigation system (Rogue Research, Cambridge, Massachusetts) tracked the participant’s position using their MRI scan if available (8 subjects) or the Montreal Neurological Institute template (Lancaster *et al* 2007). We used a Magstim D70 Remote Coil (Jali Medical, Framingham, MA) in a semi-random search for elicitation of a left first-dorsal-interosseous (LFDI) motor response. We defined RMT as the intensity that elicited a motor-evoked potential (MEP) of > ± 50 *μ*V in response to 5 of 10 single pulses (Schutter and van Honk 2006). In addition to the LFDI target, we defined a ‘parietal’ target emanating from electrode CP2, with the coil handle pointed anteriorly to align stimulation perpendicular to the main gyral axis of the superior parietal lobe.

**Figure 1.**
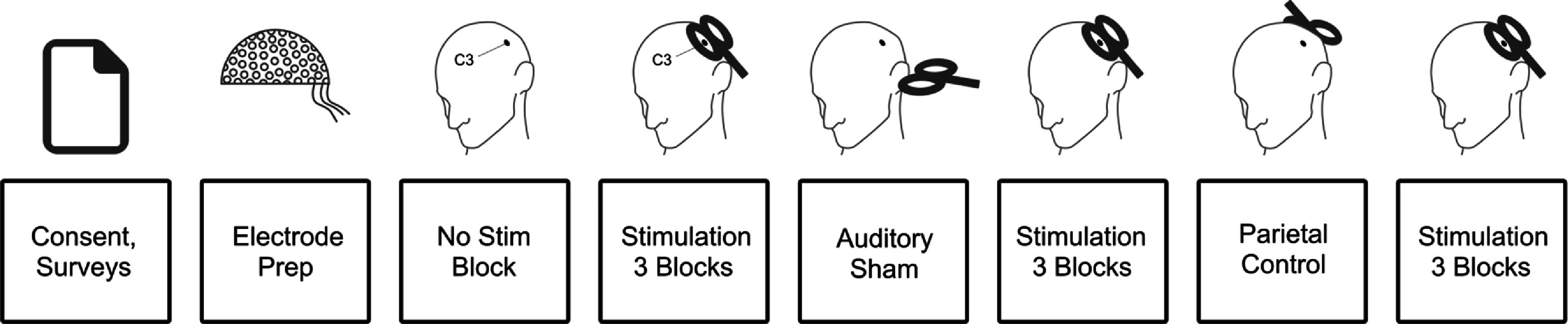
Session layout. Each box represents a session component in sequential order from left to right. The C3 electrode (center of the Laplacian montage) is depicted. Note that the stimulation target of the non-control blocks was left first-dorsal-interosseous (LFDI), not the C3 electrode, though these positions were typically very close to each other.

Participants first performed one four-minute block of eyes-open resting-state EEG (the ‘no-stim’ block). Then, in each of 12 blocks, we applied 80 single pulses of TMS while participants sat in resting-state. The 4th and 8th blocks were fixed as the ‘parietal’ and ‘auditory sham’ blocks, respectively, since these blocks required coil repositioning and provided a break for the participant. The no-stim, auditory sham and parietal conditions served as controls for the peripheral effects of stimulation. In the auditory sham block stimulation was fired into the air 2.5 cm from the left ear with the coil parallel to the floor. In the auditory sham and parietal condition intensity was 100% RMT. In the remaining ten blocks stimulation was applied to the LFDI target intensities from 10% to 100% RMT in steps of 10% (denoted as the ‘*X*% RMT block’ throughout, *X* being intensity), at counterbalanced order (figure 1). We visually monitored participants’ alertness. The minimum inter-block break was at least 1 min.

In stimulation blocks TMS was triggered by a closed-loop system which continuously monitored the EEG in real-time and fired TMS when the C3-*µ* signal amplitude met a maximum artifact threshold (in raw *µ*V) and minimum *µ*-power criterion, which were continuously adjusted to maintain a firing rate of more than one stimulation every four seconds (Zrenner *et al* 2018; see supplementary material). The minimum inter-stimulus interval (ISI) was 2 s. This ISI generally does not induce carryover effects between pulses, and prior phase-dependent studies have used ISIs in this range for studying single-pulse TEPs (Zrenner *et al* 2018, Gordon *et al* 2022). Due to the firing conditions, the jittered ISI standard deviation was 1.9 s, the jitter of which prevented frequency-based stimulation effects. Any undetected remaining accumulative effect of the single pulse stimulation would have affected all conditions equally across participants, because the condition order was randomly counterbalanced.

Stimulation was delivered by a Magstim D70 Air Film Coil positioned 1 mm above the target location using a rolling stand with boom arm (Manfrotto, Cassola, Italy). The experimenter corrected position using fine-adjustment knobs or by manually repositioning the participant’s head.

### 2.3. Electroencephalographic data processing

For each session, we recorded EEG with a 64-channel actiCAP slim cap with TMS-compatible Ag/AgCl active electrodes (Brain Vision, Morrisville, NC) mounted in an elastic mesh fabric cap according to the extended International 10-20 System. We adjusted impedances below 15 kΩ and recorded in a shielded room. We acquired EEG using Lab Streaming Layer (Swartz Center for Computational Neuroscience, UCSD, open-source available online) and the LSL-actiCHamp Connector (Brain Products, open-source available online).

We repeated several of our analyses on subsets of trials closer (±90°) to estimated Alpha peaks (‘peak-trials’) or troughs (‘trough-trials’) in the data, collectively referred to as the ‘phase-sorted’ analyses. To estimate the phase at each stimulation we used procedures similar to those described in Zrenner *et al* (2018); briefly, we cut corrupted data near the TMS pulse and used non-corrupted data to train an autoregressive (AR) model to estimate the phase at firing. We implemented this pipeline offline in Python. We estimated the accuracy of this approach by applying the same processing to trials without TMS and comparing our predictions to ground-truth. Accuracy was defined as:

\begin{align*}{\text{accuracy}} = 1 - \frac{1}{{180}}\left| {{\theta _i} - {\theta _{\text{t}}}} \right|,\end{align*}

where ${\theta _i}$ is the estimated phase for trial *i*, and ${\theta _{\text{t}}}$ is the target phase. An accuracy of 1 means that there was no phase deviation, while an accuracy of 0 means that the two angles were separated by 180°. Our accuracy was 69.24%, which is similar to previous implementations (Zrenner *et al* 2018, Madsen *et al* 2019). Other details of our implementation are included in the supplementary information.

### 2.4. Phase locking factor and transcranial evoked potential analysis methods

Phase resetting is often operationalized across trials as phase locking factor (PLF), a metric that quantifies how similar phases are across trials at the same time point relative to an event (the TMS pulse), with 0 and 1 representing no and perfect phase consistency, respectively. Randomly sampled trials will have PLF near zero unless a stimulus induces phase resets. PLF was calculated as:

\begin{align*}{\text{PLF}}\left( t \right) \triangleq \frac{1}{N}\left| {\mathop \sum \nolimits_r^{{{\tiny\unicode{x2B1A}}}} {e^{i\theta r\left( t \right)}}} \right|,\end{align*}

where *N* is the total number of trials, *r* is the *r*th trial, and *θ_r_(t)* is the instantaneous phase of trial *r* at time point *t* derived from the Hilbert transformed signal (Kitajo *et al* 2013).

We also explored how TMS and phase modulate the N100 TEP component. The negative peak latency within each participant’s grand average TEP from 75 to 125 ms was identified, and the N100 amplitude and PLF were extracted at that individualized latency.

### 2.5. Statistical analysis

**Figure 2.**
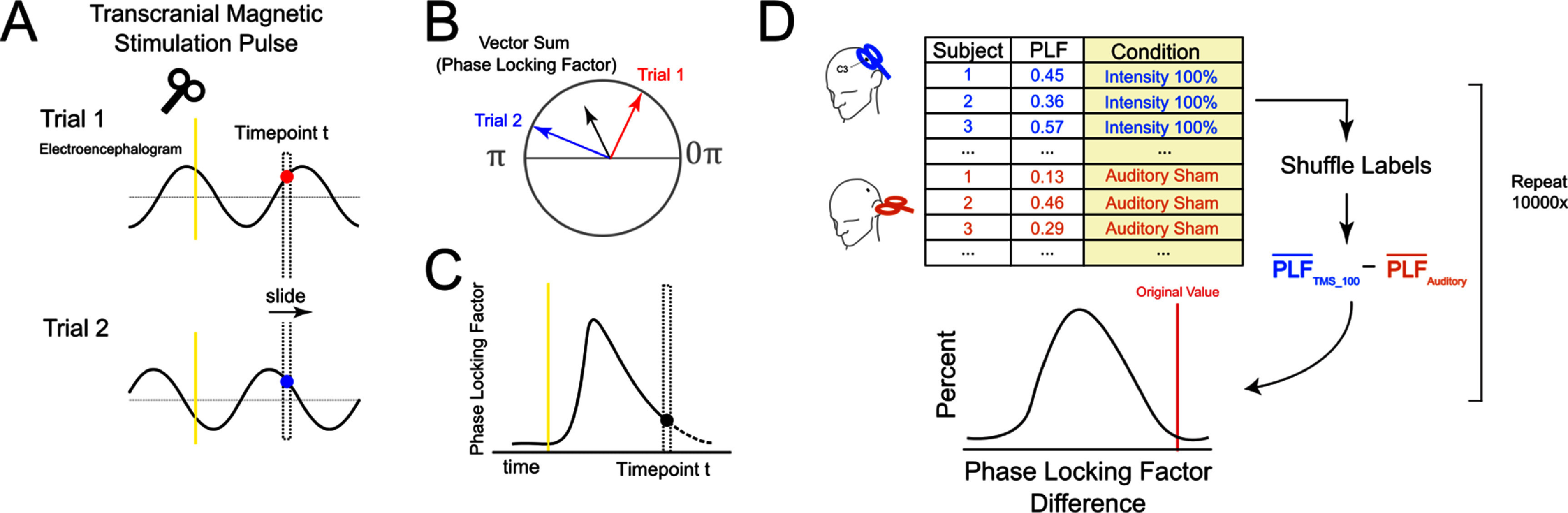
Phase locking factor (PLF) computation and permutation test. Schematic of our method for computing PLF and calculating significance, utilizing a permutation test to develop a null distribution. (A): Each recording block was epoched by time locking to stimulations. Each time point in the epoch was then considered in a sequential fashion. (B): For each time point, we computed PLF, which is the average vector sum of the instantaneous phase angle across trials. We can then draw the timecourse of PLFs, as shown in (C). We then conducted permutation tests at each time point to identify significant periods of phase locking relative to the control condition, as shown in (D). In this example, we are comparing the PLF of the 100% RMT stimulation condition to auditory sham. We shuffle the condition labels 10 000 times and compute the difference between the average PLFs of the two conditions to obtain a null distribution. The true PLF mean difference is compared to the null distribution to obtain a significance value by dividing the number of null PLF differences by the total number of permutations, and corrected by the Benjamini Hochberg false discovery rate procedure (Benjamini and Hochberg 1995).

Our main comparison identified periods of significant PLF differences between the FDI stimulation conditions at different TMS intensities and the auditory sham condition. We used a non-parametric permutation method to compare the differences in these distributions (figure 2; Good 2013). At each timepoint, we computed the PLF for each participant within each condition. We computed the average PLF across all participants for each condition, and then took the average of these values within each condition at each timepoint. The pairwise difference between these within-condition averages (100% RMT condition minus auditory sham) was the actual observed PLF difference at each timepoint. We tested the significance of these differences across time against the expected null PLF difference distribution. To compute this expected distribution, we shuffled the average PLF condition labels across participants, recomputed the average PLF across these shuffled conditions, and recomputed the pairwise differences. We performed this procedure over 10 000 permutations of the condition labels, resulting in a null distribution of PLF condition differences with 10 000 points. We computed *p* values as the number of points in the null distribution that were higher than the original computed pairwise differences, representing the chance probability of our original observed value. We did not have an *a priori* hypothesis about which timepoints would exhibit significant differences, so after computing the *p* value for each timepoint we applied a Benjamini–Hochberg false discovery rate procedure and used an adjusted *p* < 0.05 threshold for significance (Benjamini and Hochberg 1995). We applied this analysis and computed significant timepoints for the peak-trial sorted data and the trough-trial sorted data separately.

We applied a similar permutation test approach to test for a parametric relationship between PLF and TMS intensity. For each timepoint, we computed the average PLF within the participant and power level (for conditions stimulating FDI only). We then computed the Spearman correlation between PLF and TMS power level for the original data. To create a null distribution of expected Spearman correlations, we then shuffled the condition labels and recomputed the Spearman correlations. We performed this procedure over 10 000 permutations of the condition labels, resulting in a null distribution of Spearman correlations with 10 000 points. We computed *p* values identically as the previous analysis, also applying the same false discovery rate procedure.

## 3. Results

### 3.1. Phase locking factor and transcranial evoked potentials

In several conditions, PLF increased throughout the baseline period. This is an artifact of the acausal filter smoothing post-stimulation effects backward in time. Despite this artifact, baseline PLF was not significantly different between 100% RMT LFDI stimulation and either control condition.

At intensities above ∼40% RMT we observed an increase in PLF over the whole timecourse, which agrees with prior evidence that TMS above this intensity evokes brain responses and measurably influences cortical excitability (Kujirai *et al* 1993, Ilić *et al* 2002, Berger *et al* 2011). 100% RMT stimulation induced significantly larger PLF than the auditory sham condition over a wide time period (supplementary table 1). We did not observe significant C3-*µ* PLF differences between parietal site stimulation and LFDI stimulation. The spreading activation of a pulse can induce motor cortex PLF from stimulation at sites as distant as occipital cortex (Kawasaki *et al* 2014). Therefore, 100% RMT parietal stimulation likely induced some C3 PLF via multisynaptic network effects and our study may have been underpowered to observe a difference between these conditions.

### 3.2. Phase-sorted analyses

**Figure 3.**
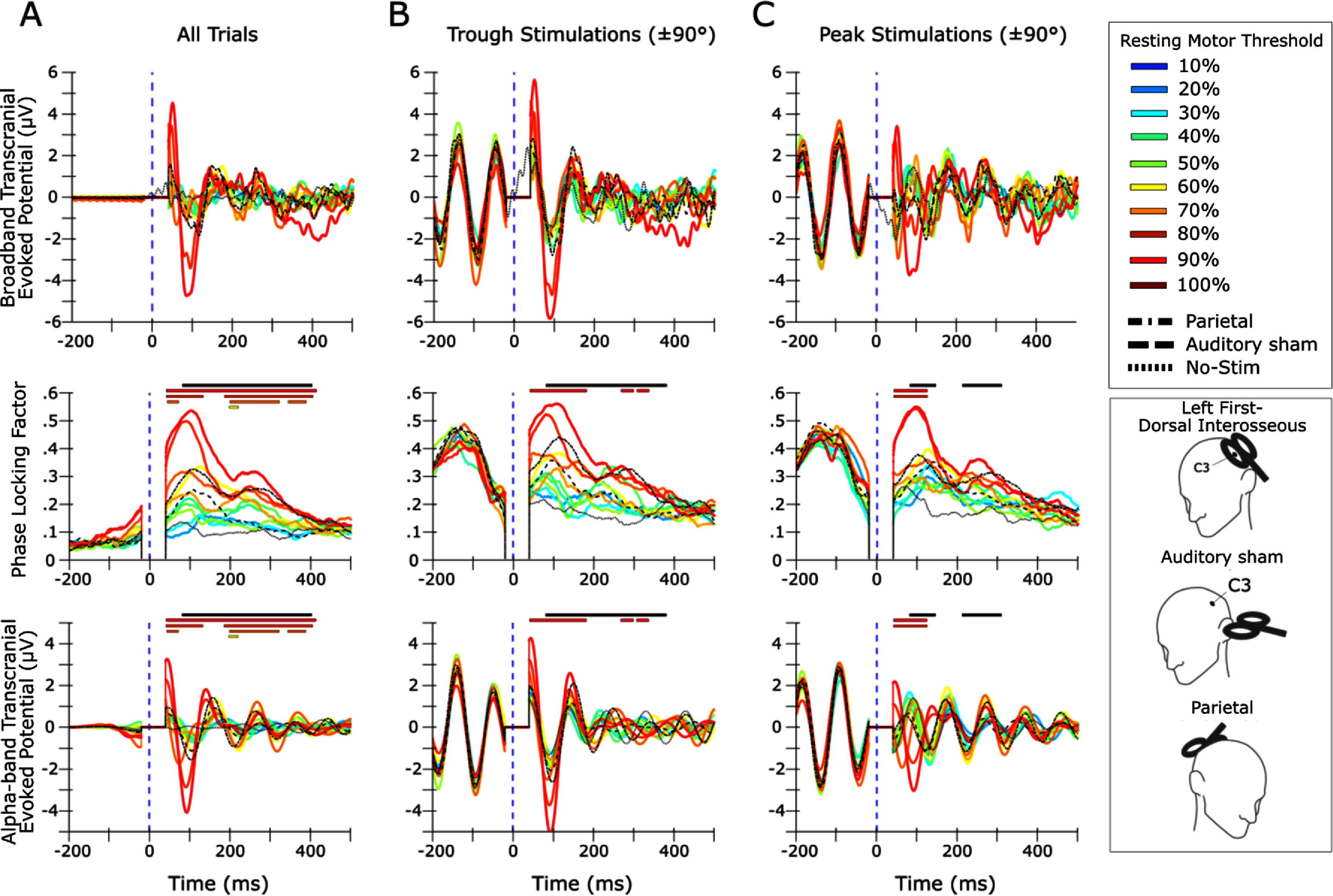
Phase-locking factor (PLF) and transcranial-evoked potential (TEP) time courses within transcranial magnetic stimulation (TMS) intensity and instantaneous phase. In each panel, the top figure shows the timecourse of C3-*µ* PLF and the bottom figure shows the C3-*µ* TEP. (A): results computed over all the trials collected within each stimulation condition. (B) and (C): results computed over the subset of trials in each condition whose AR-predicted phase at stimulation was closer to (within ±90° of) a trough or a peak, respectively. Colored traces represent intensities of left first-dorsal-interosseous (LFDI) stimulation. Black dashed traces represent the auditory sham, parietal and no-stim control conditions. Significant time periods after family-wise detection rate correction (*α* = .05) are represented by bars over each figure. Solid black significance bars represent where PLF is parametric with TMS intensity. Colored significance bars correspond to the LFDI-stim trace with the same color and represent where that LFDI stimulation intensity induced significantly greater PLF compared to auditory sham. Only power levels of 60% resting motor threshold (RMT) and above met this criterion. No intensity induced PLF significantly greater than parietal stimulation. Exact latencies of significant periods are given in supplementary table 1. Note that 90% and 100% RMT stimulation near peaks reversed the phase of Alpha-band at 100 ms, while lower intensities or stimulation nearer to troughs did not. Individual-level results are included in supplementary figures 1–4

Across participants, no difference was detected in the number of trials sorted into the peak-trials and trough-trials analyses (*p* = 0.23). The rise in PLF in the baseline period was expected for phase-sorted analyses because trial selection was predicated on phase similarity. 100% RMT induced PLF greater than auditory sham in both conditions, but not from parietal control (figure 3).

**Figure 4.**
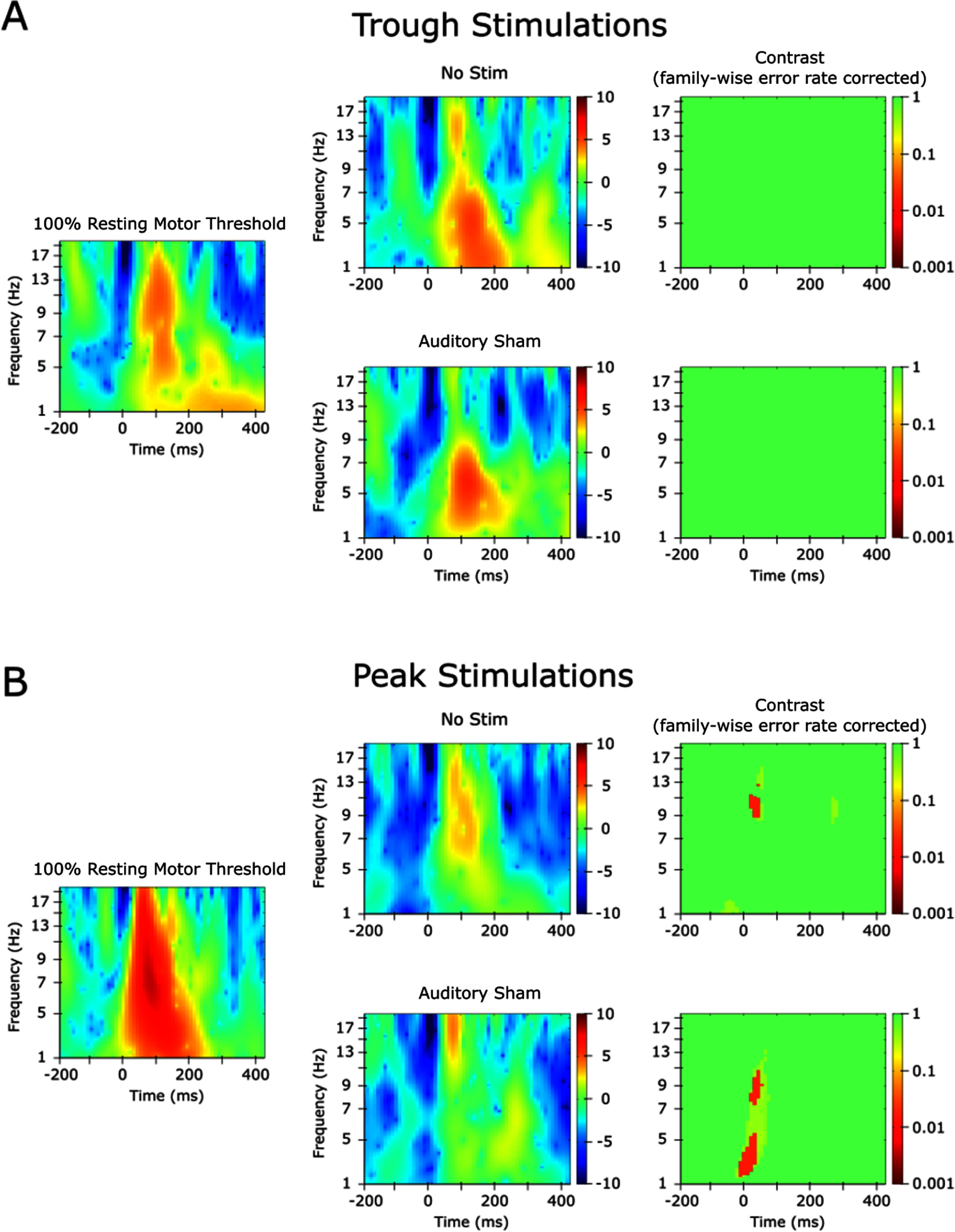
Event related spectral perturbations (ERSPs) and contrasts for 100% resting motor threshold (RMT) versus auditory sham. ERSPs reveal modulations of event-related spectral power from baseline. The ERSP is computed by calculating the time-frequency response across subjects and comparing it to the pre-stimulus interval. In each panel, the ERSP of the 100% RMT condition is shown in the left column, those of the no-stim and auditory sham conditions are shown in the middle column, and the contrast between 100% RMT and those conditions is shown in the right column. ERSPs were calculated with a shared baseline over −200–800 ms from 3 to 20 Hz using wavelets beginning at 3 cycles and increasing by 20% at each frequency. Significant clusters were identified using 2000 permutations and FDR correction. (A): 100% RMT trough stimulations did not elicit greater ERSP amplitude than control conditions. (B): 100% RMT peak stimulations elicited greater ERSP than no-stim and auditory sham stimulation in the Alpha band near the peak of the N100 component. Individual-level results are included in supplementary figures 5 and 6.

In the peak stimulation condition at intensities of 90%–100%RMT, the average broadband and Alpha-band phase was inverted at ∼100 ms. Peak stimulation event-related spectral perturbation (ERSP) also exhibited significantly greater Alpha-power than the no-stim and auditory sham conditions at this latency (figure 4). Neither of these effects were present for trough stimulation. T-tests comparing peak and trough N100 amplitudes revealed no significant differences for either broadband (*t* = − 1.148, *p* = .275) or Alpha-band (*t* = − 1.086, *p* = .300).

### 3.3. Parametric response of phase locking factor to stimulation intensity

**Figure 5.**
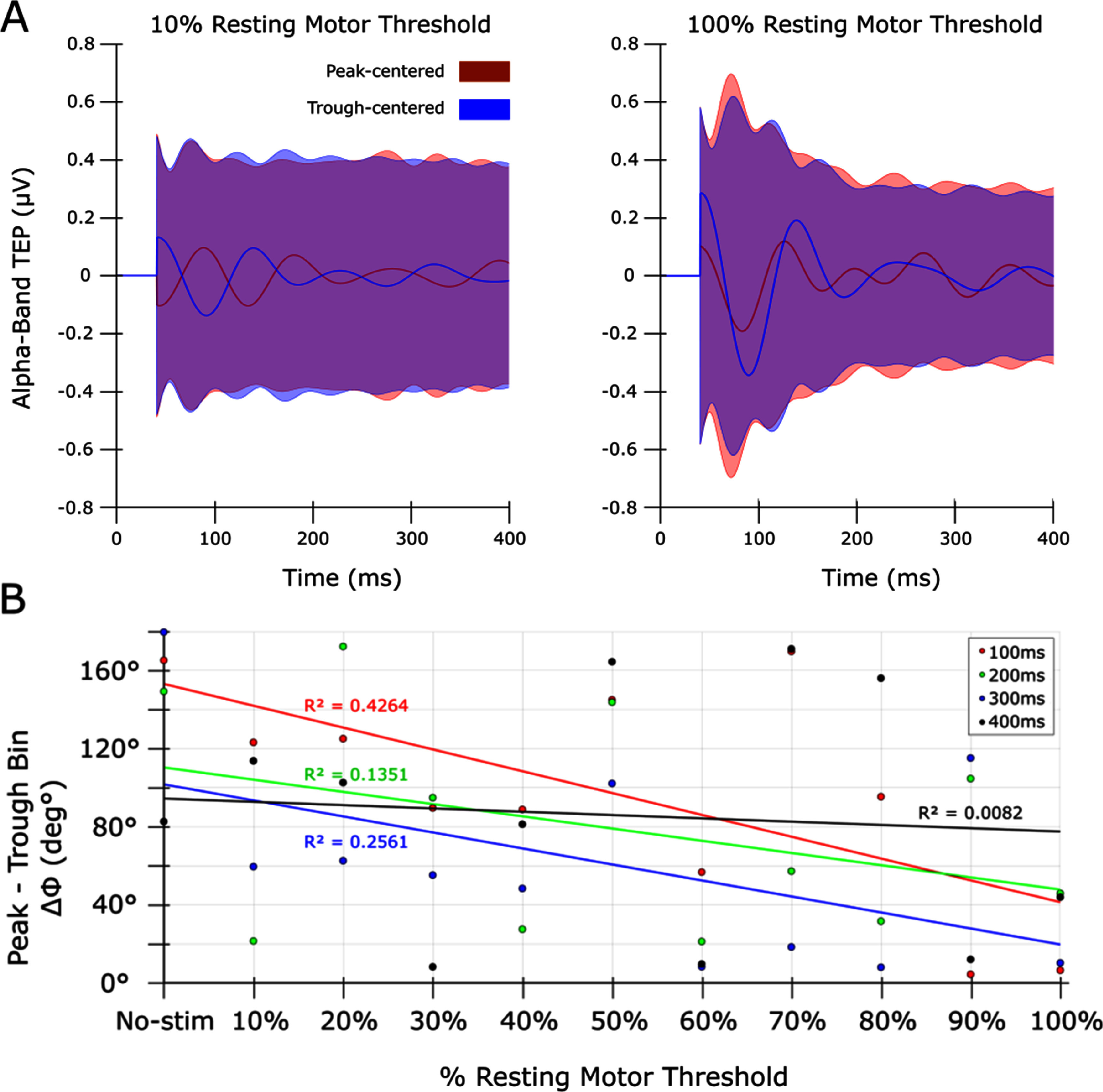
Transcranial evoked potential (TEP) phase differences by transcranial magnetic stimulation (TMS) intensity and endogenous phase. Comparison of the phase of alpha-band activity between peak- and trough-centered trials, demonstrating that stronger TMS intensity induces more similar phases, and this effect lessens at later time points. (A): TEPs of the Alpha band for peak (red) and trough (blue) stimulation bins, at selected intensities (10% and 100% resting motor threshold; RMT). Shaded regions are standard deviations. In the 10% condition (left) peak and trough bin Alpha TEPs are mostly anti-phase (ΔΦ = 170°) reflecting that TMS did not strongly affect the phase of trials in either bin. In the 100% condition (right) the phase difference between bins narrowed (ΔΦ = 10°), suggesting that TMS moved the phase of trials in the peak and trough bins closer together. (B): The ΔΦ between peak and trough stimulation bin Alpha TEPs as a function of % RMT, at 100 ms intervals up to 400 ms. A linear function is fit within each latency. The amount of resetting lessened with increasing latency, reflected by the negative slope of the fit. Individual-level results are included in supplementary figure 7.

**Figure 6.**
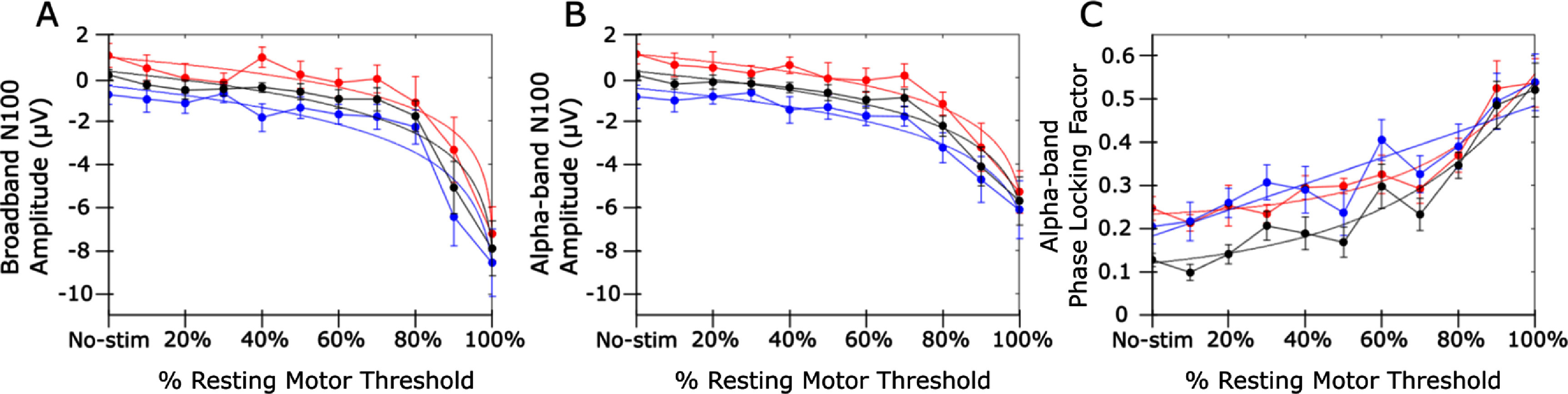
N100 transcranial evoked potential (TEP) component amplitudes and phase-locking factor (PLF) by intensity. The N100 TEP component evoked by Transcranial Magnetic Stimulation (TMS) at each TMS intensity tested. In each panel, error bars represent the standard error of the mean across participants. Black, red and blue traces represent all-trials, peak-trials and trough-trials data, respectively. For each set of trials, we tested the goodness-of-fit of a logarithmic or exponential function *versus* a linear fit (F-test, *α* = .05). (A): N100 amplitude in broadband and (B) Alpha-band as a function of TMS intensity. In both bands, the N100 generally decreased (became more negative) with increasing intensity and a logarithmic function fit all sets of trials significantly better than a linear function. (C): Alpha-band PLF as a function of TMS intensity. An exponential function fit the all-trials and peak-trials data, but not the trough data. *p* values for all fits are included in supplementary table 2. The regime of nonlinearity begins around 80%–90% resting motor threshold in all exponential fits. Individual-level results are included in supplementary figures 8 and 9.

PLF was parametric with intensity at different periods in both the all-trials, peak and trough analyses (figure 3). At early time points, the phase difference between peak and trough sorted trials was near zero after 100% RMT stimulation, supporting that complete resetting occurred (figure 5). N100 amplitude (in broadband and alpha-band) and PLF at individual N100 peak latency was nonlinearly related to TMS intensity except for trough PLF, for which the exponential fit was marginal versus a linear fit (figure 6).

## 4. Discussion

Consistent with our hypotheses, we found that the strength of *µ*-rhythm phase resetting depends on TMS intensity, and that the instantaneous phase of *µ*-Alpha at the moment of stimulation influences which phase the oscillation is reset to. We distinguished these effects from the peripheral sensory effects of TMS using auditory sham and active control site stimulation. We found that N100 amplitude was nonlinearly related to stimulus intensity, and that the N100 component underwent a full phase reset in both broadband and Alpha-band after stimulation with near-threshold intensities.

Our data reveal the nonlinear response of the motor system to intensity and suggest links to cortical excitability. Specifically, we observed TEP and PLF timecourse features coincident with the N100, an inhibitory component that likely reflects activation of GABA-*β* mediated motor inhibitory interneurons (Casula *et al* 2014, Opie *et al* 2018, Aberra *et al* 2020) and has been proposed as a marker of cortical excitability (Du *et al* 2018, Roos *et al* 2021). Saari *et al* (2018) studied this effect and found that N100 amplitude is nonlinear with intensity over a broad region centered on the motor cortex, but phase resetting is linear. In contrast we found a nonlinear effect on both phase resetting and the N100, possibly because we used a Laplacian montage focused near the site of stimulation (Hjorth 1975; figure 6). The link between the N100 and phase resetting implies that resets could play a role in how TMS modulates cortical excitability.

Our results also support that instantaneous EEG phase signals cortical excitability (Schalk 2015) and modulates phase resetting. In the motor cortex, applying TMS at the *µ*-Alpha trough as opposed to the peak results in larger MEPs (Zrenner *et al* 2018). However, at sub-threshold intensities where the MEP is not available, the TEP and PLF timecourse could contain alternative signatures of excitability. Indeed, our TEPs contained several phase-dependent features (figures 3(B) and (C)), including total phase resetting, that were obscured when averaged over instantaneous phase (figure 3(A)). Most previous studies did not examine phase dependence parametric with stimulation intensity, whereas our study allowed us to independently estimate both effects. Desideri *et al* (2019) found a modulation of N100 amplitude by phase condition at sub-threshold intensity, which we did not observe. This could be due to a total phase reset (∼180°) in the endogenous *µ*-Alpha rhythm in our data, occurring around 100 ms after peak stimulations at 80% RMT and above, in contrast to the late phase resetting (>200 ms after the pulse) found by Desideri *et al* (2019). This discrepancy could be related to our open-loop stimulation approach that sampled all of phase-space. Additionally, a study by Ding *et al* (2022) obtained different TEP and phase synchronization effects after stimulating the motor cortex at the peak and trough phases of the occipital Alpha rhythm. The timecourse of our effects generally agrees with that study, which is interesting given that the EEG source used to derive phase information was at a different location between the studies. However, Ding *et al* (2022) did not explore sub-threshold intensities, which is where we observed the transition from maintenance of the ‘expected’ endogenous phase to its opposite. We speculate that 90%–100% RMT trough stimulation reinforced endogenous phase activity, whereas the peak-stimulation induced reset faced resistance from the endogenous phase, resulting in greater *versus* attenuated TEP amplitude, respectively, though this difference was not significant. This phenomenon could be used to reset or sustain phase by modifying only stimulation timing.

Notably, there was a difference in N100 amplitude between peak- and trough-centered trials even in the ‘no stim’ condition (in which TMS was only simulated) because phase-sorting the trials leads to a persistent endogenous Alpha wave, the frequency of which coincides with the timing of the N100. This approximate amplitude difference was present at most TMS intensities, which suggests that endogenous activity coherently sums with induced TMS effects (Ding *et al* 2022). However, this does not indicate how TMS physiologically induces Alpha power. Accordingly, we additionally investigated whether spectral power was modulated after the pulse to interpret our data in the context of the additive versus resetting theories of ERP generation. PLF increases not accompanied by ERSP increases have been argued as evidence for resetting of endogenous oscillations, while simultaneous increases in both PLF and ERSP equivocally support either resetting or the activation of previously silent oscillators (Makeig *et al* 2002, Min *et al* 2007). We observed modulations of PLF at several intensities, but only observed an N100 modulation of ERSP amplitude (versus auditory sham) after 100% RMT peak stimulation (figure 4). This result generally agrees with Ding *et al* (2022). However, our finding that lower intensity stimulation induces PLF without ERSP modulations suggests that in general, TMS tends to directly reset endogenous oscillators, possibly by aligning unsynchronized ‘parallel generators’ (Thut *et al* 2011, Vernet *et al* 2013), but that at sufficient intensities and particular phases of the *µ*-rhythm new oscillators can be activated. The fact that we detected ERSP modulations from stimulations near one phase but not another suggests that a more complex phenomenon than simple coherent addition of energy is responsible for TMS effects on EEG, since simple coherent summation of waves would have led to mirrored ERSP effects across the phase bins. Furthermore, the nonlinear effect of TMS intensity on N100 amplitude also contradicts an account of simple coherent additive power. The nonlinear N100 response could potentially be related to activation of different neural populations. Prior studies have found that sub-threshold TMS primarily activates interneurons, while threshold TMS also activates pyramidal neurons. A cautious neurobiological interpretation of our results is that the contribution of low-intensity induced interneuron stimulation and high-intensity induced pyramidal neuron stimulation may contribute differentially to the N100, resulting in an overall nonlinear relationship with intensity. However, because our study did not directly measure neural activity this interpretation is only speculative.

Our study identified qualitatively different effects on the N100 and phase resetting at approximately 80% RMT. MEP studies have found that lower stimulation intensities evoke stronger phase modulated effects down to threshold (Schaworonkow *et al* 2019), but could not study sub-threshold intensities. 80% RMT has been suggested as a balance point between inhibitory and excitatory effects (Fitzgerald *et al* 2006, Berger *et al* 2011). Between 70% and 80% RMT, stimulations near peaks induced opposite average phases in our data. Therefore, we propose that the optimal intensity range for studying phase-dependent effects may be 70%–80% RMT, and add speculative support that this is a neurobiological balance point between inhibition and excitation. However, our study focuses on *µ*-Alpha, and optimal ranges could vary in other cortical areas with different intrinsic rhythms (Kawasaki *et al* 2014).

Our data suggest dynamic effects on phase later in the epoch. In general, we found a negative correlation between intensity and phase separation at all time points which waned with greater latency from the pulse (figure 5). This could be an effect of endogenous phase slippage (Freeman 2006). However, *µ*-Alpha phase did not continue unaffected after the N100 (figure 3). Instead, after peak stimulation *µ*-Alpha returned to the phase of the expected endogenous rhythm and after trough stimulation it became disrupted and out of sync (at 80%–100% RMT). Speculatively, trough TMS may have depolarized a broader population of neurons with greater inhibitory and excitatory effects, while peak TMS may have depolarized a smaller population that quickly realigned to the endogenous rhythm. A larger sample size could explore these effects for insights into the generators of *µ*-Alpha and novel phase-dependent experimental designs.

In studies such as ours, it is important to check whether peri-pulse results are contaminated by TMS peripheral evoked potentials (PEPs; Rocchi *et al* 2021). The parametric relationship we observed can only be partially attributed to the peripheral effects of stimulation as the PLF driven by our auditory sham condition (100% RMT) was lower than that of even our moderate (60% RMT) active stimulation conditions (figure 3). We spaced the coil away from the head, which prevented bone-conducted artifacts entirely. Thus, our PLF results exceed the ceiling of potential auditory effects. Auditory sham stimulation PLF returned to the post-stimulation floor at around 225 ms, suggesting that no auditory PEP effects were present after this point (figure 3). Studies argue that scalp muscle artifacts are weak compared to auditory PEPs (Rocchi *et al* 2021) especially at non-lateral sites and after 40–60 ms (Mutanen *et al* 2013, Rogasch *et al* 2017). We removed most of this early period (0–40 ms) before filtering to ensure removal of the strongest early TMS artifacts that could affect PLF before filtering.

### 4.1. Limitations

It is possible that a T1 image for each participant could marginally improve our LFDI thresholding procedure (Caulfield *et al* 2022). While we selected our target, active comparison, and negative control conditions to isolate sources of neurally-induced PLF, some peripheral and somatosensory effects could have influenced our between-condition results. Multimodal methods and direct neural recordings could be helpful to disambiguate this. Because our auditory sham intensity was not matched within condition, we may not have statistically detected real cortical phase resetting at lower intensities. Since our parietal site did not evoke an MEP, we could not target it using the same procedure as at M1. Furthermore, we were unable to distinguish cortico-cortical contributions of parietal stim from peripheral ones, which made this condition difficult to interpret. Furthermore, because estimating phase requires a high-SNR signal, we were unable to explore frequencies besides *µ*-Alpha. Our study focused on the effects of sub-threshold stimulation, but it is important for future studies to more fully explore the phase and intensity-dependent effects of supra-threshold stimulation which may be dominated by the response of different neural populations, in order to better target this stimulation to achieve specific brain effects.

## 5. Conclusion

We showed that TMS phase resetting depends on intensity and the phase of EEG. Our study detected nonlinear oscillatory responses that speculatively might signal the sub-threshold activation of different neural populations, and added evidence about the likely additive versus resetting source and phase-dependence of these effects. We hope that our results will inform the development of more effective and targeted rTMS protocols that explicitly aim to induce resetting or additive effects, including in the context of oscillatory brain state. Most importantly, our data showed that the direction of EEG phase, as opposed to its magnitude, is only modulated when near-threshold stimulation is applied nearer to the peak of the endogenous rhythm. This effect could be utilized to test phase-behavior relationships with experimental protocols that vary only in their stimulus timing. Our results also suggest that between 70% and 80% RMT may be the most sensitive regime for detecting phase-dependent effects. Future research could investigate the sensitivity of EEG phase resetting as a predictor of the strength of cortical excitability modulation throughout the cortex, which could potentially be exploited for control and enhancement of cognitive functions.

## Data Availability

The data that support the findings of this study are openly available at the following URL/DOI: https://doi.org/10.17605/OSF.IO/D3XQN (Brian *et al* 2024). Data will be available from 09 April 2025.
